# Betaine based organ preservation solution alleviates renal allograft I/R injury by protecting endothelial cells

**DOI:** 10.1093/rb/rbag064

**Published:** 2026-03-26

**Authors:** Mingxing Yu, Shuo Li, Xu Duan, Yiran Wang, Tao Ming, Shaohua Wu, Xihe Wang, Zhongyang Shen, Xunfeng Zou, Deling Kong, Tingting Lan

**Affiliations:** Research Institute of Transplant Medicine, Tianjin First Central Hospital, School of Medicine, Nankai University, Tianjin 300192, China; State Key Laboratory of Medicinal Chemical Biology, College of Life Science, Nankai University, Tianjin 300350, China; Research Institute of Transplant Medicine, Tianjin First Central Hospital, School of Medicine, Nankai University, Tianjin 300192, China; State Key Laboratory of Medicinal Chemical Biology, College of Life Science, Nankai University, Tianjin 300350, China; Research Institute of Transplant Medicine, Tianjin First Central Hospital, School of Medicine, Nankai University, Tianjin 300192, China; State Key Laboratory of Medicinal Chemical Biology, College of Life Science, Nankai University, Tianjin 300350, China; Research Institute of Transplant Medicine, Tianjin First Central Hospital, School of Medicine, Nankai University, Tianjin 300192, China; State Key Laboratory of Medicinal Chemical Biology, College of Life Science, Nankai University, Tianjin 300350, China; Research Institute of Transplant Medicine, Tianjin First Central Hospital, School of Medicine, Nankai University, Tianjin 300192, China; Clinical Laboratory, Tianjin First Central Hospital, School of Medicine, Nankai University, Tianjin 300192, China; Research Institute of Transplant Medicine, Tianjin First Central Hospital, School of Medicine, Nankai University, Tianjin 300192, China; State Key Laboratory of Medicinal Chemical Biology, College of Life Science, Nankai University, Tianjin 300350, China; Research Institute of Transplant Medicine, Tianjin First Central Hospital, School of Medicine, Nankai University, Tianjin 300192, China; Department of General Surgery, Tianjin First Central Hospital, School of Medicine, Nankai University, Tianjin 300192, China; Research Institute of Transplant Medicine, Tianjin First Central Hospital, School of Medicine, Nankai University, Tianjin 300192, China; State Key Laboratory of Medicinal Chemical Biology, College of Life Science, Nankai University, Tianjin 300350, China; Research Institute of Transplant Medicine, Tianjin First Central Hospital, School of Medicine, Nankai University, Tianjin 300192, China

**Keywords:** organ preservation solution, betaine, anti-apoptotic, ischemia-reperfusion injury

## Abstract

Static cold preservation remains a cornerstone of transplantation, and high-performance organ preservation solutions, sharing the same goal as regenerative biomaterials, are indispensable for facilitating the regeneration and functional recovery of organs following transplantation. In this study, we developed a betaine-based organ preservation solution (BOPs), combined with the optimization of osmotic pressure components and energy support components. In the endothelial cell inflammation model, BOPs exhibited robust antioxidative and anti-apoptotic effects. Across multiple stages of kidney transplantation, including static cold storage, early reperfusion and the post-*in situ* transplantation period, BOPs demonstrated organ preservation efficacy comparable to that of the University of Wisconsin (UW) solution. Owing to its favorable efficacy and cost-effectiveness, BOPs hold broad market prospects.

## Graphical Abstract

**Figure:**
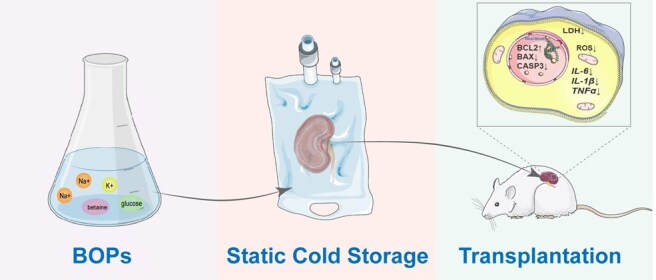

## Introduction

With the intensifying global aging population, the incidence of end-stage organ failure is steadily increasing, making it a major public health challenge. Organ transplantation remains the only curative therapy, yet its application is severely constrained by the shortage of donor organs. Improving donor organ quality and extending preservation time have therefore become critical to enhancing transplantation outcomes. Since organ preservation directly determines graft viability and postoperative function, optimization of preservation technology is a central focus. Normothermic machine perfusion (NMP) has emerged as a promising approach, but its high cost, technical complexity and stringent requirements for dynamic regulation currently limit its widespread use [1, 2]. In contrast, static cold storage (SCS) is still the clinical mainstay due to its simplicity and low cost [3]. However, SCS is associated with cellular energy depletion and microcirculatory damage, which markedly increase the risk of primary non-function (PNF) [4, 5].

To overcome the limitations of SCS, recent research has concentrated on refining preservation solutions, targeting three main aspects: osmotic regulation, antioxidant defense and energy substrate supplementation. Clinically used solutions include the University of Wisconsin (UW) solution, Histidine-Tryptophan-Ketoglutarate (HTK) solution and Hypertonic Citrate Adenine (HCA) solution [6, 7]. Although differing in formulation, all aim to mitigate cell injury by suppressing metabolism, stabilizing the ionic environment and reducing reactive oxygen species (ROS) [8]. UW, considered the gold standard, significantly prolongs preservation time through its hyperosmotic environment, potassium-rich composition and inclusion of metabolic inhibitors (e.g. adenosine and raffinose), which collectively preserve cell integrity [9]. Nevertheless, its high viscosity can cause uneven perfusion, its potassium content predisposes to reperfusion arrhythmias and its high cost (>$500 per unit) restricts its clinical application [6]. By comparison, HTK has lower viscosity, a simpler composition and is far more cost-effective (1/5–1/3 the cost of UW), providing economic and handling advantages. However, its preservation capacity is shorter: kidneys exhibit microcirculatory dysfunction beyond 24 h of preservation and its low sodium content poses a risk of post-transplant hyponatremia [10, 11]. HCA, developed using domestic raw materials, further reduces costs (1/10 the cost of UW) [12], while the relatively high concentration of magnesium sulfate in its formulation tends to precipitate at low temperatures. Such precipitation, both in the preservation solution and within blood vessels, can induce tissue damage. Additionally, the formulation lacks an effective buffer pair, leading to unstable pH values that are insufficient to prevent cellular acidification during long-term organ preservation. Current cold preservation solutions still exhibit limitations in biocompatibility, ice crystal control and preservation efficacy and thus cannot fully meet the diverse, complex preservation requirements in clinical practice. Therefore, further research is warranted to develop cold preservation solutions with enhanced performance and broader versatility.

Recent advances in organ preservation have shifted the paradigm in preservation solution design from broad metabolic suppression [13] to targeted cytoprotection, with increasing emphasis on cellular specificity and functional resilience. Within the kidney, endothelial cells have emerged as central mediators in this context, acting as the frontline defense during ischemia-reperfusion (I/R) injury and transplantation [14, 15]. Throughout the transplantation process—including kidney retrieval, cold storage and reperfusion—endothelial cells endure repeated stress from warm ischemia, cold ischemia and reoxygenation. These insults disrupt mitochondrial electron transport, leading to electron leakage and superoxide ($O_{2}^{-}$) generation, while xanthine metabolism releases uric acid and additional ROS. The accumulation of ROS damages parenchymal cell DNA, activates the JNK/p38 MAPK pathway, upregulates pro-apoptotic mediators such as Caspase-3 (CASP3) and reduces the BCL2/BAX ratio, thereby driving apoptosis [15–18]. Concurrently, ischemic endothelial cells release damage-associated molecular patterns, which activate TLR4/NF-κB signaling and trigger robust expression of pro-inflammatory cytokines, such as tumor necrosis factor-α (TNF-α), interleukin-1β (IL-1β) and interleukin-6 (IL-6) [19, 20]. These amplify inflammation and propagate injury to neighboring glomerular, tubular and parenchymal cells. Collectively, oxidative stress and inflammation within renal endothelial cells represent central mechanisms driving parenchymal apoptosis, underscoring the importance of developing next-generation preservation solutions with targeted endothelial protection [21].

Betaine is a natural alkaloid widely distributed in both animals and plants, possessing multiple biological functions, including serving as a methyl donor, regulating osmotic pressure and exerting anti-oxidative, anti-inflammatory and anti-apoptotic effects. It has demonstrated considerable potential in the management of metabolic diseases, neuroprotection and anti-aging. Previous studies [22] have shown that betaine delays renal aging by inhibiting TBK1 kinase activity and blocking the IRF3/NF-κB inflammatory pathway. In aged mouse models, oral administration of betaine significantly improved renal function indicators, such as serum creatinine and urea nitrogen and attenuated renal fibrosis [23]. *In vitro*, betaine pretreatment effectively suppressed inflammatory responses in macrophages, renal cortical epithelial cells, mesenchymal progenitor cells and aortic endothelial cells [24, 25]. Specifically, betaine reduced the expression and secretion of TNF-α, IL-6 and IL-8; lowered ROS levels; downregulated inflammation-related genes; and prevented lipopolysaccharide (LPS)-induced monocyte adhesion [22, 26]. These findings strongly support the anti-inflammatory and renoprotective effects of betaine, highlighting its unique role in mitigating renal aging. Moreover, betaine enhances nitric oxide (NO) synthesis, promotes vasodilation, inhibits platelet aggregation and endothelial inflammation and markedly improves endothelial cell survival [27]. In the field of cryopreservation, betaine has been validated as an effective osmotic regulator, reducing cryoinjury in a wide range of cells and tissues, including sperm [28], vascular tissue, muscle [29] and spleen [30]—thereby underscoring its potential as a cellular protectant [31]. However, its application in cold storage solutions, particularly for kidney preservation, has not yet been explored.

Building on our previous findings that betaine-containing solutions mitigate cold ischemic injury in the liver [32], betaine was used as the key component, combined with the optimization of osmotic pressure components and energy support components; the cold storage solution named betaine-based organ preservation solution (BOPs) was developed. In this study, the antioxidative and anti-apoptotic effects of betaine were investigated in an endothelial cell inflammation model. Moreover, the kidney was selected as a representative organ and comprehensive evaluations were conducted at various stages, including SCS, early reperfusion simulated by NMP and the initial post-*in situ* transplantation period. Our findings demonstrate that BOPs provides organ preservation efficacy comparable to UW and exhibits superior potential in osmotic regulation and antiapoptotic protection.

## Materials and methods

### Experimental animals

Male Sprague–Dawley (SD) rats, 6–8 weeks old and weighing 200–220 g, were housed under standard conditions with free access to food and water in a 12 h light/dark cycle. Animals were obtained from Beijing HFK Bioscience Co., Ltd (Beijing, China; Certificate number: SCXK Jing 2019-0008). All animal experiments were approved by the Institutional Animal Care and Use Committee of Nankai University (license number: 2021-SYDWLL-000393).

### Cell culture and inflammation induction

Human umbilical vein endothelial cells (HUVECs) were purchased from ATCC (Manassas, VA, USA). Cells were cultured in endothelial cell medium (ECM) (1001, ScienCell Research, USA) supplemented with 10% fetal bovine serum (FBS) and 50 µg/mL penicillin/streptomycin in a humidified incubator (5% CO_2_, 37°C). Cells at 80–90% confluence were used for experiments. For experimental induction of inflammation, HUVECs were seeded into six-well plates and cultured to 80–90% confluence. Three experimental groups were established: a control group, an LPS-induced model group and a betaine treatment group. After washing with phosphate-buffered saline (PBS), cells in the betaine group were pretreated with 10 mM betaine for 1 h. Subsequently, 1 µg/mL LPS (L8880, Solarbio, Beijing, CN) was added to both the model group and the betaine treatment group to induce inflammatory stimulation. Following 6 h of stimulation, the cells were harvested for subsequent analysis.

### Oxygen-glucose deprivation/reoxygenation cold storage model

The preparation method of BOPs has been elaborated in a previous study [32]. Additionally, certain components have been optimized, and the details of these optimizations are presented in Table 1, along with a comparison of the components between BOPs and UW solutions. HUVECs were seeded in 6-well, 48-well or 96-well plates at densities of 2 × 10^5^, 5 × 10^4^, and 1 × 10^4^ cells per well, respectively. When cells reached 80–90% confluence, they were washed with PBS and treated with one of the following: basal medium, UW solution, BOPs [(+) BOPs] or betaine-deficient BOPs [ (−) BOPs]. Plates were transferred to a hypoxia chamber, where the air was displaced with nitrogen for 10–15 min. Following 24 h of cold storage, cells were rewarmed in complete medium under normoxic conditions (5% CO_2_, 37°C). Cell supernatants and pellets were collected at 0, 2 and 6 h post-rewarming for lactate dehydrogenase (LDH) assay, immunofluorescence staining or real-time quantitative polymerase chain reaction (RT-qPCR) analysis, flow cytometry analysis and western blot analysis, respectively.

**Table 1:** Components of the BOPs solution and UW solution (mmol/L).^a^

Components	UW	BOPs
Betaine	–	15
HES200 (g/L)	50	50
Raffinose	30	–
Glucuronic acid	–	55
Na^+^	30	115
K^+^	125	42
Mg^2+^	5	5
Cl^−^	–	5
${\text{SO}}_{4}^{2-}$	5	35
${\text{H}}_{2}{\text{PO}}_{4}^{-}/{\text{HPO}}_{4}^{2-}$	25	25
Sucrose	–	10
D-glucose	–	5
DOG	–	7
Glycine	–	5
Allopurinol	1	–
Glutathione	3	–
Adenosine	5	–
pH	7.4	7.4
Osmolarity	320	320

a HES200, hydroxyethyl starch with molecular weight 200 kDa; DOG: 2-deoxy-D-glucose; Osmolarity was measured and showed in mOsm/kg.

### Cellular biological behavior assessment

Following oxygen-glucose deprivation/reoxygenation (OGD/R), cell viability was measured using the Cell Counting Kit-8 (CCK-8) assay. Briefly, 10 µL of CCK-8 (MA0218, Meilunbio, Dalian, CN) was added to each well of 96-well plates (per 100 µL medium) and incubated at 37°C for 1–2 h. Absorbance was measured at 450 nm using a microplate reader. For phalloidin (RM02836, ABclonal, Wuhan, CN) and TUNEL (MPC250704, Servicebio, Wuhan, CN) staining, cells were fixed with 4% paraformaldehyde (PFA) for 30 min, permeabilized with 0.2% Triton X-100 for 30 min and blocked with 5% bovine serum albumin (BSA) at 37°C for 1 h. Cells were then incubated with phalloidin or TUNEL dye for 2 h, followed by 4',6-diamidino-2-phenylindole (DAPI) counterstaining. Fluorescent images were acquired using a confocal microscope (FV100, Olympus, Tokyo, Japan). For mitochondrial membrane potential assessment, JC-1 staining (MA0338, Meilun, Dalian, CN) was applied. Cells were incubated with 5 µM JC-1 working solution at 37°C for 15–30 min in the dark. After washing with staining buffer, cells were observed under a confocal microscope (excitation wavelength [λex] = 488 nm; green emission wavelength [λem]: 525 nm; red λem: 590 nm). Intracellular ROS levels were measured using 2',7'-dichlorodihydrofluorescein diacetate (DCFH-DA) (S105S, Beyotime, Shanghai, CN) staining. Cells were incubated with 10–20 µM DCFH-DA at 37°C for 20 min in the dark. After washing, green fluorescence was detected via confocal microscopy (λex = 488 nm, λem = 525 nm).

### Kidney harvesting and cold preservation

Rats were anesthetized via intraperitoneal injection of 10% chloral hydrate (0.2 mL/100 g body weight) combined with Zoletil 50 (0.02 mL/100 g body weight) in early experiments. For later experiments, adhering to updated animal welfare guidelines, rats were anesthetized with intraperitoneal Zoletil 50 (0.02 mL/100 g), followed by isoflurane inhalation during surgery. Following the abdominal incision, the left kidney was exposed, renal vessels were carefully dissected and the renal artery was cannulated. Kidneys were flushed with heparinized saline until complete blood clearance, then allocated to experimental groups for preservation. Animals were euthanized via CO_2_ inhalation. Kidneys were assigned to one of five groups: (i) fresh control, (ii) saline-treated model, (iii) UW solution preservation, (iv) (+) BOPs preservation and (v) (−) BOPs preservation. All kidneys were stored at 4°C for 24 h. Supernatants were collected for LDH assays, and kidney tissues were harvested for histological analysis, RT-qPCR or NMP assay. To evaluate organ edema, tissue samples were weighed immediately after retrieval (wet weight) and then dried to constant mass at 65°C for 48 h (dry weight). The dry-to-wet weight ratio was calculated as an indicator of tissue water retention; lower ratios indicate more severe tissue edema following cold storage.

### *In vitro* perfusion of kidneys using NMP

Kidneys were connected to a prewarmed (37°C) perfusion circuit containing oxygenated perfusate, as described previously [33]. A tetrafluoride cannula (0.3–0.6 mm diameter) was inserted into the renal artery. Perfusion pressure was maintained at 40 mmHg for 1 h. Following perfusion, perfusate samples and kidney tissues were collected for histological and molecular analyses.

### Kidney transplantation

Donor kidneys were preserved at 4°C for 12–16 h using UW, (+) BOPs and (−) BOPs solutions. Renal transplantation was performed on recipient rats, with an end-to-end anastomosis of the left renal vessels and ureter using 10-0 silk sutures. To create a functional graft model, the contralateral right kidney was removed. Before closing the abdomen, a single dose of meloxicam, dexamethasone and gentamicin was administered for postoperative analgesia, anti-inflammation and antimicrobial prophylaxis. In the UW versus BOPs preservation groups, blood samples were collected preoperatively and on postoperative Days 1, 3, 5 and 7 to measure serum creatinine (SCr), blood urea nitrogen (BUN) and cytokines including IL-6, IL-1β and TNF-α; kidneys were harvested on postoperative Day 7. For the subgroup examining betaine’s role, kidneys preserved with (+)/(−) BOPs were harvested on postoperative Day 2, and corresponding blood samples were collected to determine SCr, with urine samples collected for urinary creatinine (UCr) analysis.

### Immunofluorescent staining

Kidneys were harvested and fixed with 4% PFA, dehydrated, paraffin-embedded and sectioned at 5 µm. For immunofluorescence, antigen retrieval was conducted using sodium citrate buffer, followed by permeabilization with 0.2% Triton X-100, blocking with 5% BSA and incubation with primary antibodies anti-BAX (GB114122, Servicebio, Wuhan, CN; 1:200) and anti-ICAM1 (ab282575, Abcam, Cambridge, UK; 1:400) overnight at 4°C. After being rinsed three times with PBS, tissues were incubated with corresponding secondary antibodies Alexa Fluor 647 (A11029, Invitrogen, Carlsbad, USA) or Alexa Fluor 488 (A11008, Invitrogen, Carlsbad, USA). Fluorescent images were acquired using a confocal microscope (FV1000, Olympus).

### Histology

H&E of the kidneys were performed according to our previous study [33].

### Histopathological evaluation

For pathological scoring, acute tubular necrosis (ATN) was assessed based on the following criteria: renal tubular epithelial cell swelling, vacuolization, brush border loss, luminal dilation, cast formation and necrosis. Injury severity was graded on a 0–4 scale (0 = no injury; 1 = mild; 2 = moderate; 3 = severe; 4 = very severe).

The scoring system incorporated key parameters associated with renal transplant injury, including tubulointerstitial inflammation, tubular atrophy, interstitial fibrosis, glomerulitis and peritubular capillaritis. Each parameter was evaluated based on the Banff grading criteria, ranging from 0 to 3 points, with higher scores reflecting greater tissue damage. The overall score was then calculated to quantify the extent of pathological injury in each group.

### RT-qPCR assessment

Total RNA was extracted from tissues or cells using TRIzol reagent (ZOMANBIO, Beijing, China) according to the manufacturer’s protocol. Complementary DNA (cDNA) was synthesized using the First Strand cDNA Synthesis Kit (ZOMANBIO, Beijing, China). Quantitative polymerase chain reaction (qPCR) was performed with Bestar qPCR Master Mix on a LightCycler 96 (Roche, Mannheim, Germany), following the kit instructions. Relative gene expression levels were calculated using the 2^−ΔΔCT method, with glyceraldehyde-3-phosphate dehydrogenase (*GAPDH*) as the internal reference gene. Primer sequences were designed and synthesized by Sangon Biotech (Shanghai, China), and details are provided in Table 2.

**Table 2:** PCR primer sequences.^a^

Gene	Forward primer (5′-3′)	Reverse primer (5′-3′)
h*TNF-α*	GAGGCCAAGCCCTGGTATG	CGGGCCGATTGATCTCAGC
h*IL-6*	CCTGAACCTTCCAAAGATGGC	TTCACCAGGCAAGTCTCCTCA
h*IL-1β*	GCACCTGTACGATCACTGAACTG	CACTTGTTGCTCCATATCCTGTCC
h*BAX*	AAGGTGCCGGAACTGATCAG	GTCTTGGATCCAGCCCAACA
h*BCL2*	GAACTGGGGGAGGATTGTGG	CATCCCAGCCTCCGTTATCC
h*CASP3*	TGCATACTCCACAGCACCTG	TCTGTTGCCACCTTTCGGTT
h*GAPDH*	GGAGCGAGATCCCTCCAAAAT	GGCTGTTGTCATACTTCTCATGG
r*TNF-α*	ACTGAACTTCGGGGTGATCG	GCTTGGTGGTTTGCTACGAC
r*IL-6*	CTTCCAGCCAGTTGCCTTCTTG	TGGTCTGTTGTGGGTGGTATCC
r*IL-1β*	GCACAGTTCCCCAACTGGTA	ACACGGGTTCCATGGTGAAG
r*BAX*	GGAGACACCTGAGCTGACCTTG	CATCGCCAATTCGCCTGAGAC
r*BCL2*	TACGAGTGGGATACTGGAGATGAAG	TCAGGCTGGAAGGAGAAGATGC
r*CASP3*	CGGTATTGAGACAGACAGTGGAAC	GCGGTAGAGTAAGCATACAGGAAG
r*GAPDH*	TGGTGAAGCAGGCATCTGAG	TGCTGTTGAAGTCGCAGGAG

a Gene abbreviations include a species-specific prefix: ‘r’ denotes rat and ‘h’ denotes human.

### Enzyme-linked immunosorbent assay evaluation

Serum IL-6, IL-1β and TNF-α were measured using rat-specific enzyme-linked immunosorbent assay (ELISA) kits (IL-6: Cat. RMK0570; IL-1β: Cat. RM17833; TNF-α: Cat. RM17824, all from ABclonal, Wuhan, China), following the manufacturer’s protocols. Serum samples were diluted 1:10–1:100 in assay buffer to ensure absorbance values within the linear range of the standard curve. A total of 50 µL diluted serum or standard was added to pre-coated wells and incubated at 37°C for 1 h, followed by washing 3× with PBST (0.05% Tween 20 in PBS). Diluted HRP-conjugated detection antibody (100 µL/well) was then added and incubated at 37°C for 1 h. TMB substrate (50 µL/well) was added and incubated in the dark at 37°C for 15 min, after which the reaction was stopped. Absorbance at 450 nm was recorded using a microplate reader, and cytokine concentrations were calculated from standard curves.

### Flow cytometry assay

To perform apoptosis analysis using Annexin V/7-AAD staining (A8515020, Yeasen, Shanghai, CN), cells were initially washed once in PBS and subsequently in 1× Binding Buffer. The cells were then resuspended in 1× Binding Buffer at a concentration of 1–5 × 10^6 cells/mL. A total of 5 μL of fluorochrome-conjugated Annexin V was added to 100 μL of the cell suspension. The mixture was incubated for 10–15 min at room temperature. Following incubation, cells were washed and resuspended in 200 μL of 1× Binding Buffer, and 5 μL of 7-AAD Viability Staining Solution was added. The samples were analyzed by flow cytometry (BD FACSAria II, San Jose, USA) immediately. The total apoptosis rate was subsequently calculated.

### Western blot

Total protein was extracted from kidney tissues using RIPA lysis buffer on ice, supplemented by a protease and phosphatase inhibitor cocktail to ensure protein integrity. Protein concentrations were quantitatively assessed using the bicinchoninic acid (BCA) assay, following the manufacturer’s protocol. Subsequently, equal amounts of protein lysates were subjected to 10% sodium dodecyl sulfate-polyacrylamide gel electrophoresis (SDS-PAGE) for separation and transferred onto nitrocellulose membranes. Membranes were blocked with 5% nonfat milk in Tris-buffered saline containing Tween-20 (TBST) for 2 h at room temperature to prevent nonspecific binding. Following blocking, membranes were incubated overnight at 4°C with primary antibodies targeting BAX (GB114122, Servicebio, Wuhan, CN; 1:800) and Anti-GAPDH-HRP (ZB15004, Servicebio, Wuhan, CN; 1:1000). The next day, membranes were washed with TBST and incubated with HRP-conjugated secondary antibodies (BA1058, BOSTER, Wuhan, CN) at room temperature for 2 h. Protein bands were visualized using a Bio-Rad chemiluminescence detection system, and their relative gray values were quantified with ImageJ software, using GAPDH as the internal reference for normalization.

### Blood plasma clotting analysis

Blood samples were collected from SD rats (sham and BOPs groups) on the second day post-surgery and mixed with 3.8% sodium citrate as an anticoagulant. After resting at room temperature for 30 min, samples were centrifuged at 3000 rpm for 10 min to separate the plasma. Four coagulation parameters—prothrombin time (PT, R01002, Rayto, Shenzhen, CN), activated partial thromboplastin time (APTT, R01102, Rayto, Shenzhen, CN), thrombin time (TT, R01202, Rayto, Shenzhen, CN) and fibrinogen (FIB, R01302, Rayto, Shenzhen, CN)—were measured using an automated coagulation analyzer following the manufacturer’s protocols. All assays were conducted in five replicates to ensure accuracy and reproducibility.

### Platelet SEM observation

For platelet isolation, whole blood was centrifuged at 200 g for 10 min to obtain the upper platelet-rich plasma (PRP). Three experimental groups were included: platelets from the sham group, platelets from the BOPs group and thrombin-activated platelets as a positive control. For activation, thrombin was added to PRP at 1 U/mL and incubated at room temperature for 15 min. The PRP was then centrifuged at 800 g for 10 min to pellet the platelets. The platelet pellet was gently washed twice with PBS and allowed to adhere to glass slides for 15 min. Samples were fixed with 2.5% glutaraldehyde at 4°C overnight. After dehydration through a graded ethanol series, specimens were dried, sputter-coated with gold and examined under a scanning electron microscope (SEM, QUANTA 200, FEI, Hillsboro, USA) to evaluate morphological changes in the platelets.

### Statistical analysis

Data were analyzed using GraphPad Prism 9 (GraphPad Software, La Jolla, CA) and expressed as mean ± SD. Comparisons were performed by unpaired Student’s *t* test or one-way ANOVA with Dunnett’s *post hoc* test, where appropriate. Statistical significance was considered at *P *< 0.05.

## Results

### Betaine protects mitochondrial function and reduces endothelial injury in cold preservation

**Figure 1:**
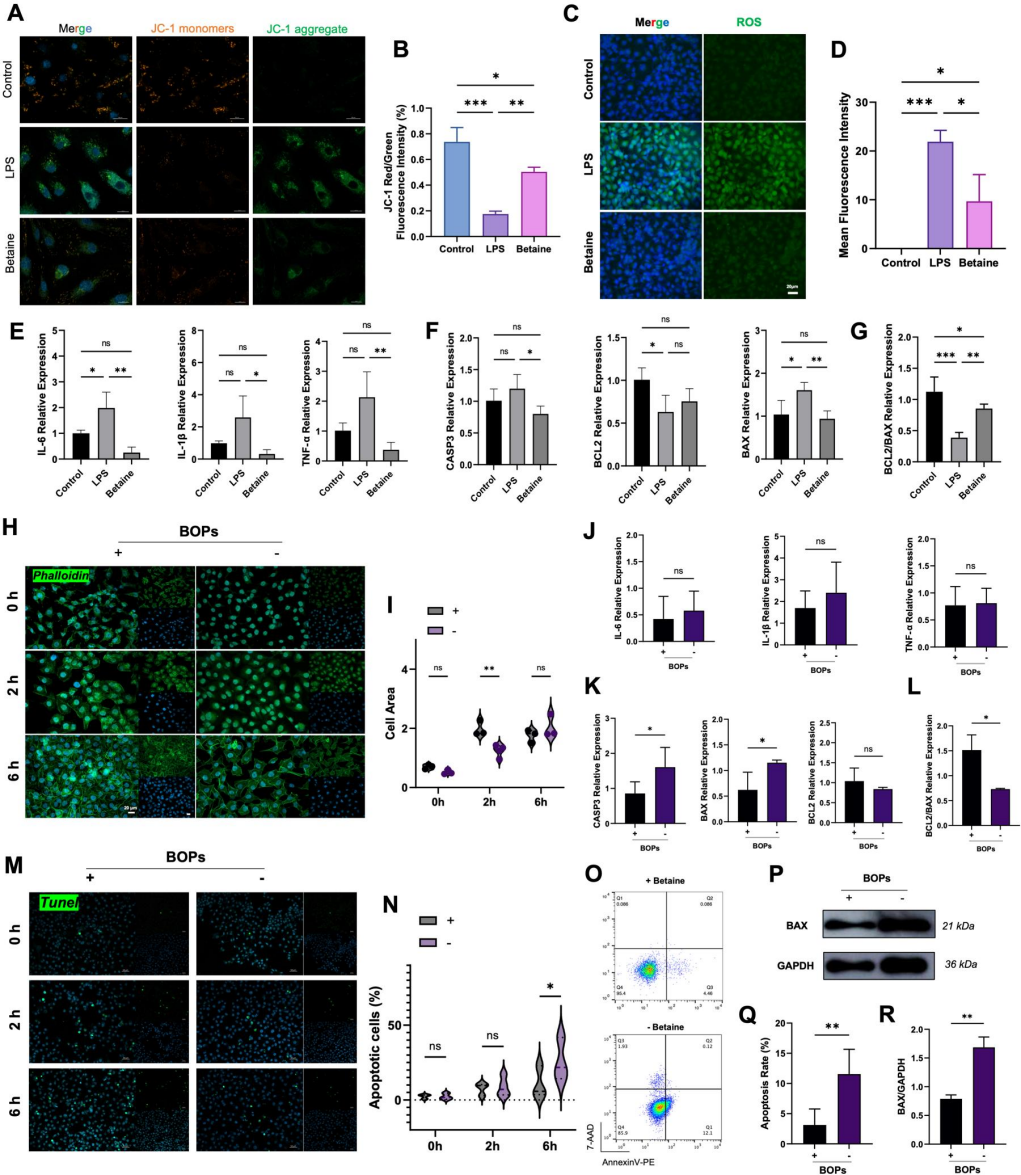
Betaine preserves mitochondrial function and mitigates endothelial injury during cold preservation. (**A**) JC-1 staining of HUVECs stimulated with LPS for 6 h to assess ΔΨm depolarization (scale bar: 20 μm). (**B**) Quantification of ΔΨm depolarization by calculating the JC-1 aggregates/JC-1 monomers fluorescence intensity ratio. (**C**) Confocal microscopy images showing intracellular ROS levels in LPS-stimulated HUVECs (DCFH-DA staining) and (**D**) quantification of mean fluorescence intensity. (**E**) Relative mRNA expression levels of pro-inflammatory cytokines *IL-6*, *IL-1β* and *TNF-α*. (**F**) Relative mRNA expression levels of apoptosis-related genes *CASP3*, *BCL2* and *BAX*. (**G**) The BCL2/BAX ratio (a lower ratio indicates a stronger apoptotic tendency). (**H**) Phalloidin staining of the cytoskeleton (scale bars: 20 μm) and (**I**) quantification of cell area. (**J**) Relative mRNA expression levels of *IL-6*, *IL-1β* and *TNF-α* in the HUVEC cold storage model. (**K**) Relative mRNA expression levels of *CASP3*, *BAX* and *BCL2*, and (**L**) the BCL2/BAX ratio. (**M**) Representative TUNEL immunofluorescence images and (**N**) quantification of apoptotic HUVECs following OGD/R cold storage and rewarming. (**O**) Flow cytometry analysis of AV/AAD expression in the HUVECs cold storage model in BOPs includes (+) or excludes (−) betaine and (**Q**) the quantification of apoptosis rate (%). (**P** and **R**) Immunoblot analysis of BAX protein in the HUVECs cold storage model in BOPs includes (+) or excludes (−) betaine. Data are presented as mean ± SD, *n* = 3. *P < 0.05, **P < 0.01, ***P < 0.001, ns: not significant.

To investigate the protective role of betaine against oxidative stress-induced endothelial injury, an inflammatory injury model was established using LPS-stimulated HUVECs. Mitochondrial membrane potential (ΔΨm) depolarization, a hallmark of early apoptosis, was evaluated by JC-1 staining (Figure 1A). In the LPS group, markedly increased green fluorescence (monomeric form) was observed, indicating severe ΔΨm loss. In contrast, both the control group and the betaine-pretreated group exhibited predominant red fluorescence (J-aggregates), suggesting intact mitochondrial membrane potential. Quantitative analysis of the red/green fluorescence ratio further confirmed that betaine effectively prevented LPS-induced ΔΨm depolarization (Figure 1B), thereby reducing early apoptosis. Excessive ROS accumulation is a key driver of mitochondrial dysfunction. DCFH-DA staining demonstrated that LPS stimulation significantly elevated ROS levels in HUVECs, whereas betaine pretreatment markedly suppressed this increase (Figure 1C and D), indicating its potent antioxidative effect. At the transcriptional level, RT-qPCR analysis revealed that betaine significantly inhibited LPS-induced upregulation of mRNA expression of pro-inflammatory cytokines *IL-6*, *IL-1β* and *TNF-α* (Figure 1E). Furthermore, betaine reduced the expression of the pro-apoptotic genes *BAX* and *CASP3*, while upregulating the anti-apoptotic gene *BCL2*, resulting in an increased BCL2/BAX ratio (Figure 1F and G). These findings suggest that betaine exerts cytoprotective effects by modulating mitochondrial function.

To further explore its translational potential in organ preservation, betaine was incorporated into a self-prepared preservation solution (BOPs) and a HUVEC OGD/R cold storage model was established to mimic I/R injury. Morphological assessment showed that although cells in both groups underwent shrinkage during cold storage, they recovered a stretched morphology after rewarming, with no obvious morphological differences (Figure S1). We performed cytoskeletal staining with phalloidin on HUVECs treated with betaine-containing (+) BOPs and betaine-free (−) BOPs after cold storage for 0, 2 and 6 h and quantitatively analyzed cell spreading area. Results showed that after 2 h of cold storage, the (+) BOPs-treated group exhibited significantly higher cell spreading than the (−) BOPs-treated group (Figure 1H and I). Similarly, no significant differences in *IL-6*, *IL-1β* and *TNF-α* expression were observed after 2 h of rewarming (Figure 1J). However, cells preserved in (−) BOPs exhibited upregulated *BAX* and *CASP3* expression, whereas the (+) BOPs group maintained a higher BCL2/BAX ratio and lower *CASP3* expression (Figure 1K and L). Further TUNEL staining assessed apoptosis in cold-preserved cells (Figure 1M), and the proportion of apoptotic cells was quantified (Figure 1N). Data indicated that (−) BOPs treatment significantly increased endothelial cell apoptosis levels. These findings were further validated by Annexin V/7-AAD dual-staining flow cytometry (Figure 1O and Q) and pro-apoptotic protein BAX western blot analysis (Figure 1P and R). Collectively, these results demonstrate that betaine not only alleviates LPS-induced oxidative stress and inflammation but also reduces endothelial apoptosis under cold storage conditions, highlighting its potential as a functional additive for organ preservation.

### BOPs provides endothelial protection comparable to UW

**Figure 2:**
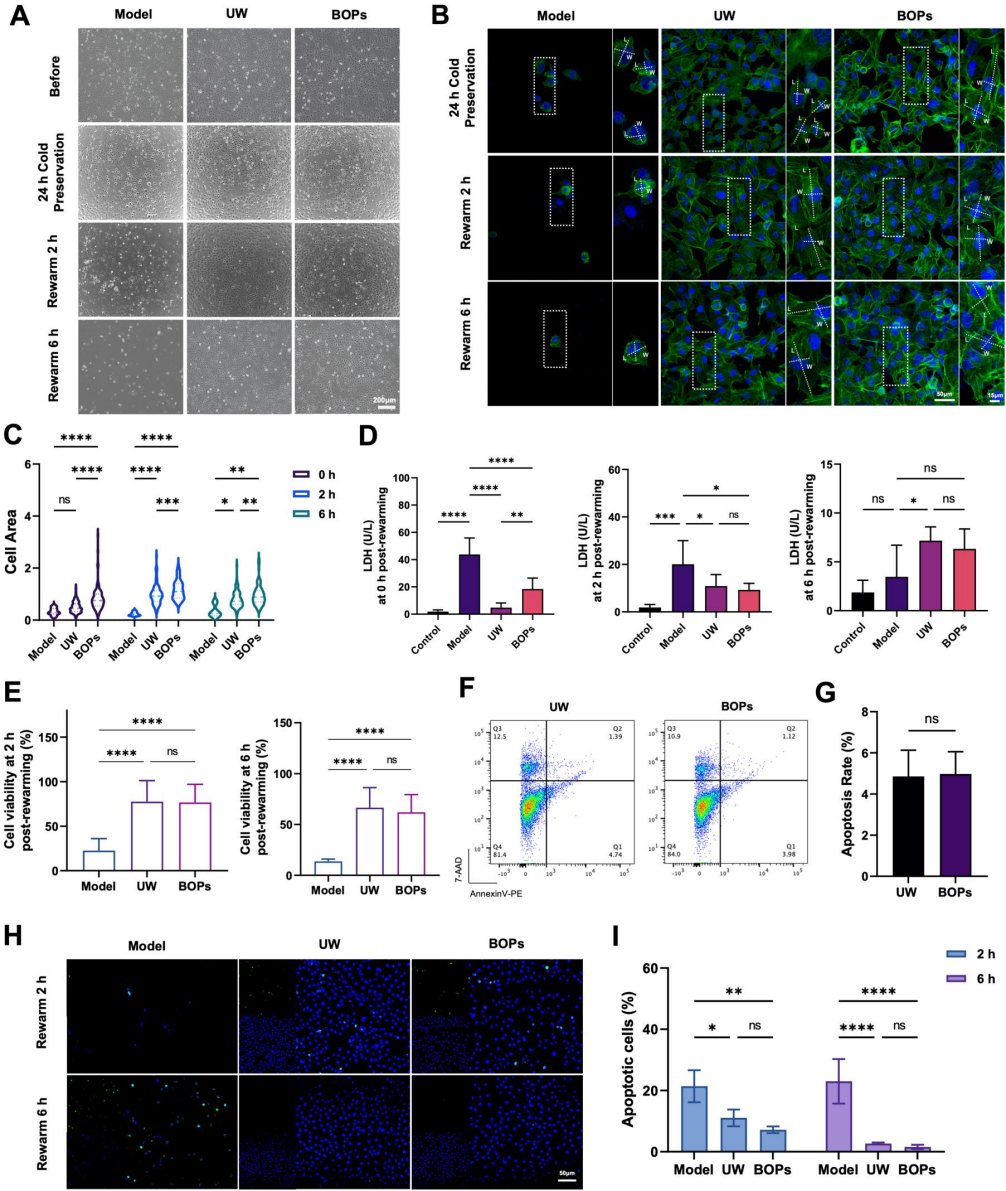
BOPs affords endothelial protection comparable to UW. (**A**) Representative light-field images of HUVECs after OGD/R cold storage and rewarming (scale bar: 200 μm). (**B**) Phalloidin staining of the cytoskeleton (scale bars: 50 and 15 μm) and (**C**) quantification of cell area. (**D**) LDH levels in cell culture supernatants measured after cold storage and at 2 and 6 h post-rewarming. (**E**) Cell viability at 2 and 6 h after rewarming, assessed by CCK-8 assay. (**F** and **G**) Flow cytometry analysis of AV/AAD expression in the HUVECs cold storage model in BOPs or UW. (**H** and **I**) Representative TUNEL immunofluorescence images and quantification of apoptotic HUVECs following OGD/R cold storage and rewarming. Data are presented as mean ± SD, *n* = 3. *P < 0.05, **P < 0.01, ***P < 0.001, ****P < 0.0001, ns: not significant.

To evaluate the preservation efficacy of BOPs, systematic analyses were performed at the endothelial cell level. Light-field microscopy (Figure 2A) and phalloidin staining (Figure 2B) revealed that during cold storage, the majority of cells in the untreated model group detached and died due to insufficient cryoprotection. In contrast, both the UW and BOPs groups showed markedly improved cellular morphology. Notably, cell shrinkage was less pronounced in the BOPs group compared to UW. Upon rewarming for 2 and 6 h, BOPs-treated cells exhibited greater spreading capacity, although cell viability did not differ significantly between the BOPs and UW groups (Figure 2C and E).

To further assess cell damage, LDH release was quantified. During cold storage, LDH release in the BOPs group was higher than in the UW group (Figure 2D). This phenomenon may be attributed to the unique metabolic properties of BOPs compared to the UW solution. Unlike the UW solution, which strongly suppresses metabolic activity, BOPs does not entirely inhibit cellular metabolism. Instead, it allows for a minimal level of metabolic activity even under hypothermic conditions. We suggest that this residual metabolic activity is a critical factor contributing to the increased levels of LDH observed during cold preservation. The model group released massive LDH during cold storage but showed a decline after rewarming, likely due to extensive cell death. In the BOPs group, LDH levels decreased progressively post-rewarming (18.43 U/L during cold storage, 9.29 U/L at 2 h and 6.33 U/L at 6 h). In contrast, the UW group displayed an opposite trend, with LDH levels increased after rewarming (4.77 U/L during cold storage, 10.88 U/L at 2 h) before decreasing (7.17 U/L at 6 h) (Figure 2D). Annexin V/7-AAD double-stained flow cytometry results (Figure 2F and G) showed that the early apoptosis rate in the UW group was 1.39%, while the late apoptosis rate was 4.74%. In the BOPs group, the rates of early and late apoptosis were 1.12 and 3.98%, respectively. Statistical analysis indicated no significant difference in the total apoptosis rate between the two groups. TUNEL staining further demonstrated extensive apoptosis in the model group, whereas apoptosis was markedly reduced in both BOPs and UW groups, with no significant difference between them (Figure 2H and I). These results indicate that BOPs provides cryoprotection and apoptosis inhibition comparable to the established UW, thereby supporting its potential as a cost-effective and effective alternative for preserving endothelial cells during cold storage.

### BOPs demonstrates anti-apoptotic potential during isolated kidney cold storage

**Figure 3:**
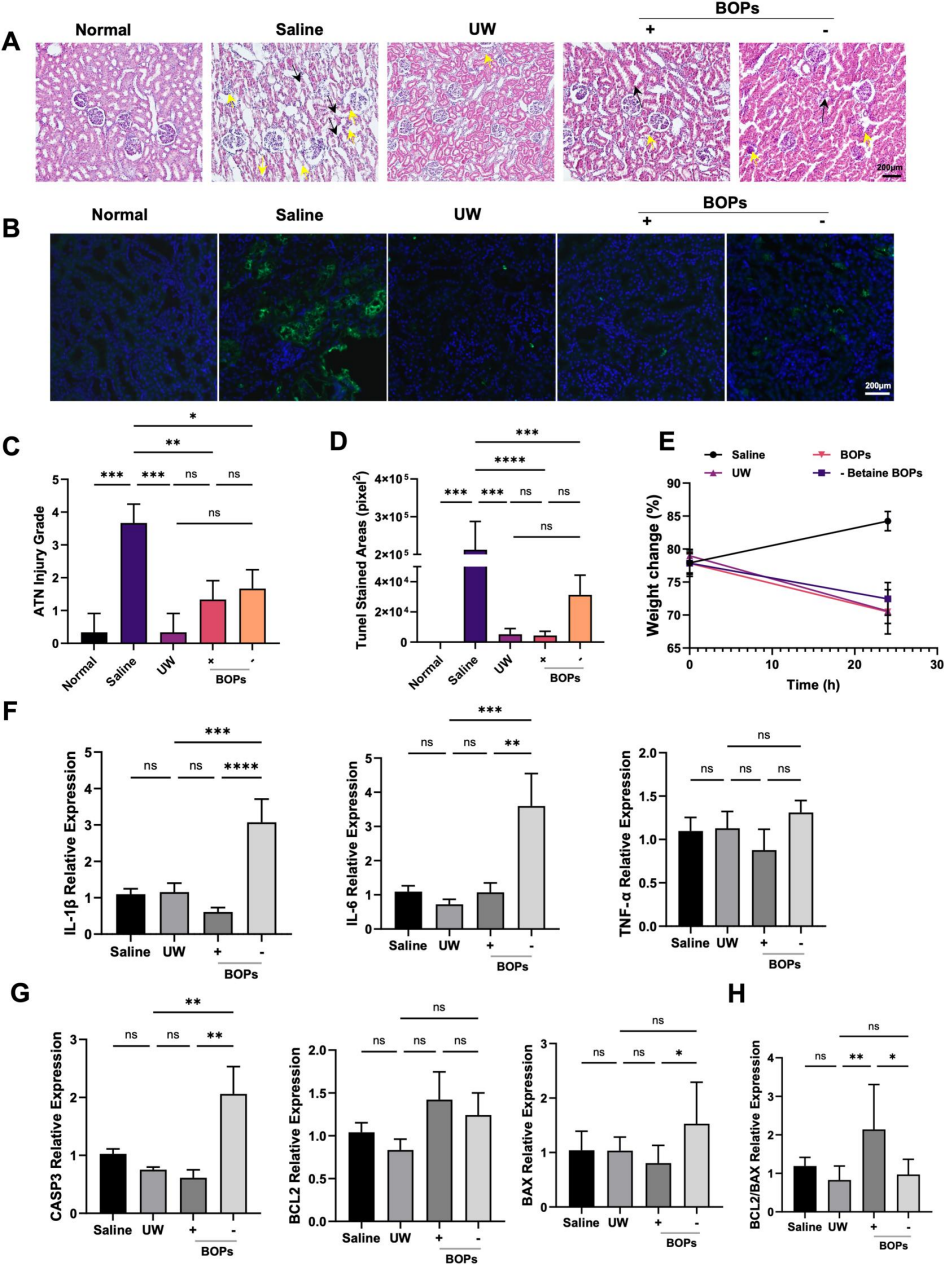
BOPs demonstrates superior anti-apoptotic potential during isolated kidney cold storage. (**A**) Representative H&E staining of isolated kidneys stored at 4°C for 24 h (scale bar: 200 μm) and (**B**) representative immunofluorescence images of apoptotic cells in cold-preserved kidneys (scale bar: 200 μm). (**C**) Quantification of ATN scores. **(D)** Quantification of apoptotic cells in cold-preserved kidneys. (**E**) Kidney weight changes before and after cold storage and rewarming. (**F**) Relative mRNA expression of inflammatory cytokines *IL-6*, *IL-1β* and *TNF-α* in kidney tissues after cold storage. (**G**) Gene expression of apoptosis-related genes *BCL2*, *BAX* and *CASP3* and (**H**) calculation of the BCL2/BAX ratio. Data are presented as mean ± SD, *n* = 6. *P < 0.05, **P < 0.01, ***P < 0.001, ****P < 0.0001, ns: not significant.

To further assess the protective effect of the (±) BOPs at the organ level, we employed an isolated rat kidney model subjected to SCS in (±) BOPs, UW solution or normal saline (model group) at 4°C for 24 h. Histopathological, apoptotic and molecular indices were subsequently evaluated. Light microscopy (Figure 3A) revealed that normal kidneys exhibited intact renal tubular epithelial cells with preserved brush borders, an absence of degeneration or necrosis, regular lumens and no dilatation or cast formation. After 24 h of cold storage in saline, renal tissues showed extensive tubular injury characterized by epithelial swelling, vacuolization (yellow arrows) and massive brush border loss (black arrows). In the UW group, tubular epithelial cells appeared shrunken with occasional edema, widened interstitial spaces and partial brush border detachment. In contrast, kidneys preserved in (±) BOPs displayed only mild cell shrinkage, limited edema, minimal brush border loss and no evidence of cast formation or necrosis. Compared to the (+) BOPs group, the number of dead cells in the (−) BOPs group increased. According to the ATN scoring system, no significant difference in tissue damage was observed between the UW and (±) BOPs groups (Figure 3C), suggesting comparable structural preservation. TUNEL staining revealed a markedly higher apoptosis rate in the saline group, whereas both UW and (±) BOPs groups exhibited only scattered positive cells, with no significant difference between them, even though the (−) BOPs group exhibited a tendency toward increased apoptotic cell count (Figure 3B and D).

To evaluate tissue edema, kidney wet-to-dry weight ratios were measured. Compared with saline, both UW and BOPs significantly reduced tissue swelling (Figure 3E, Figure S2). Water loss analysis indicated a higher water loss ratio in the UW group (28.23%) compared with the (+) BOPs group (24.27%) and the (−) BOPs group (25.9%), although the difference was not statistically significant. Combined with H&E staining, this finding suggested relatively greater interstitial fluid retention in the UW group. At the molecular level, mRNA levels of pro-inflammatory genes (*IL-1β*, *IL-6* and *TNF-α*) did not differ significantly among the Saline, UW and (+) BOPs groups during cold storage (Figure 3F), indicating minimal inflammatory activation. In the (−) BOPs group, the expression levels of the genes *IL-1β* and *IL-6* were markedly upregulated. We propose that the lack of betaine, a pivotal functional constituent, exacerbates cellular apoptosis and necrosis during cold preservation, subsequently causing heightened inflammatory responses. Conversely, in kidney tissues preserved with normal saline, the expression levels of inflammatory genes were comparatively low. We conjecture that this phenomenon results from most cells advancing to the late stages of necrosis, thereby causing a downregulation of gene transcription and expression. Moreover, *CASP3* and *BAX* expression was significantly increased in the (−) BOPs group (Figure 3G), while *BCL2* expression showed no group differences. The BCL2/BAX ratio was significantly higher in the (+) BOPs group compared with UW and (−) BOPs (Figure 3H). Collectively, these findings indicate that BOPs preserves tissue integrity, reduces edema and suppresses apoptosis more effectively than UW, particularly by modulating apoptosis-related signaling, supporting its potential as a novel organ preservation solution.

### BOPs preserves renal structural integrity during reperfusion

**Figure 4:**
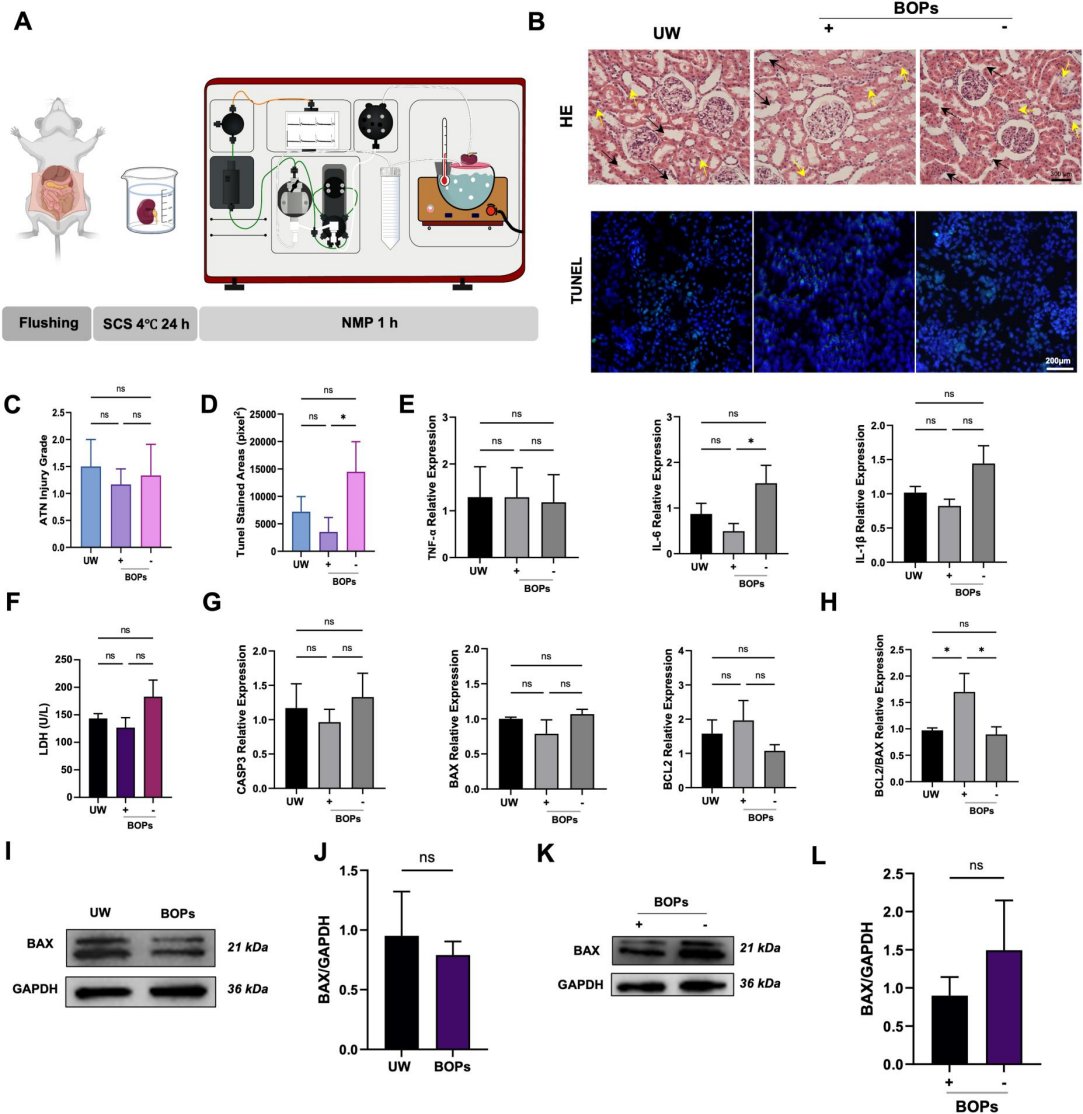
BOPs preserves renal structural integrity during reperfusion. (**A**) Schematic illustration of kidney procurement and connection to the NMP system. (**B**) Representative H&E staining and TUNEL staining images of kidney tissues after 1 h of *ex vivo* perfusion (scale bars: 50 μm). (**C** and **D**) Quantitative analysis of ATN scores and TUNEL-positive cells. (**E**) Gene expression of pro-inflammatory cytokines *IL-6*, *IL-1β* and *TNF-α* in kidney tissues after NMP. (**F**) LDH levels measured in the perfusate after 1 h of NMP. (**G**) Gene expression of apoptosis-related genes *CASP3*, *BAX* and *BCL2* and (**H**) calculation of the BCL2/BAX ratio. (**I** and **J**) Immunoblot analysis of BAX protein after 1-h extracorporeal perfusion of kidneys preserved in UW or (+) BOPs solution. (**K** and **L**) Immunoblot analysis of BAX protein after 1-h extracorporeal perfusion of kidneys preserved in (±) BOPs solution. Data are presented as mean ± SD, *n* = 6. *P < 0.05, **P < 0.01, ***P < 0.001, ns: not significant.

To model physiological and pathological events in the early post-transplantation phase, we established an *ex vivo* NMP system [33] (Figure 4A). Kidneys were subjected to 24 h of SCS at 4°C in saline, UW solution or (±) BOPs, followed by 1 h of reperfusion under 40 mmHg perfusion pressure. H&E staining revealed tubular epithelial swelling and necrosis in the UW group (Figure 4B). Kidneys preserved in (+) BOPs exhibited relatively intact tubular structures, while the (−) BOPs group exhibited cellular necrosis and structural disruption. TUNEL staining demonstrated comparable numbers of apoptotic cells between the UW and (+) BOPs groups (Figure 4D), suggesting similar anti-apoptotic effects during reperfusion. However, the number of apoptotic cells in the (−) BOPs group was significantly higher than that in the (+) BOPs group. Assessment of LDH release in the perfusate showed slightly higher levels in the (−) BOPs group, implying greater cell membrane damage (Figure 4F). RT-qPCR revealed no significant intergroup differences in *IL-1β* and *TNF-α* expression; however, the *IL-6* gene expression level in (−) BOPs was significantly higher than that in the (+) BOPs (Figure 4E). Importantly, consistent with the cold storage findings, (+) BOPs exhibited more favorable regulation of apoptosis: although *BAX*, *BCL2* and *CASP3* expression levels did not differ statistically (Figure 4G), the BCL2/BAX ratio was significantly higher in the BOPs group (Figure 4H), reflecting reduced apoptotic susceptibility. Immunoblotting results showed no statistically significant differences in the expression levels of the pro-apoptotic protein BAX between the BOPs group (Figure 4I and J) and the UW group, nor between the (+) BOPs group and the (−) BOPs group (Figure 4K and L). These results suggest that (+) BOPs better sustain cell survival signaling pathways, conferring enhanced anti-apoptotic potential during reperfusion.

### BOPs promotes functional recovery and improves early prognosis after kidney transplantation

**Figure 5:**
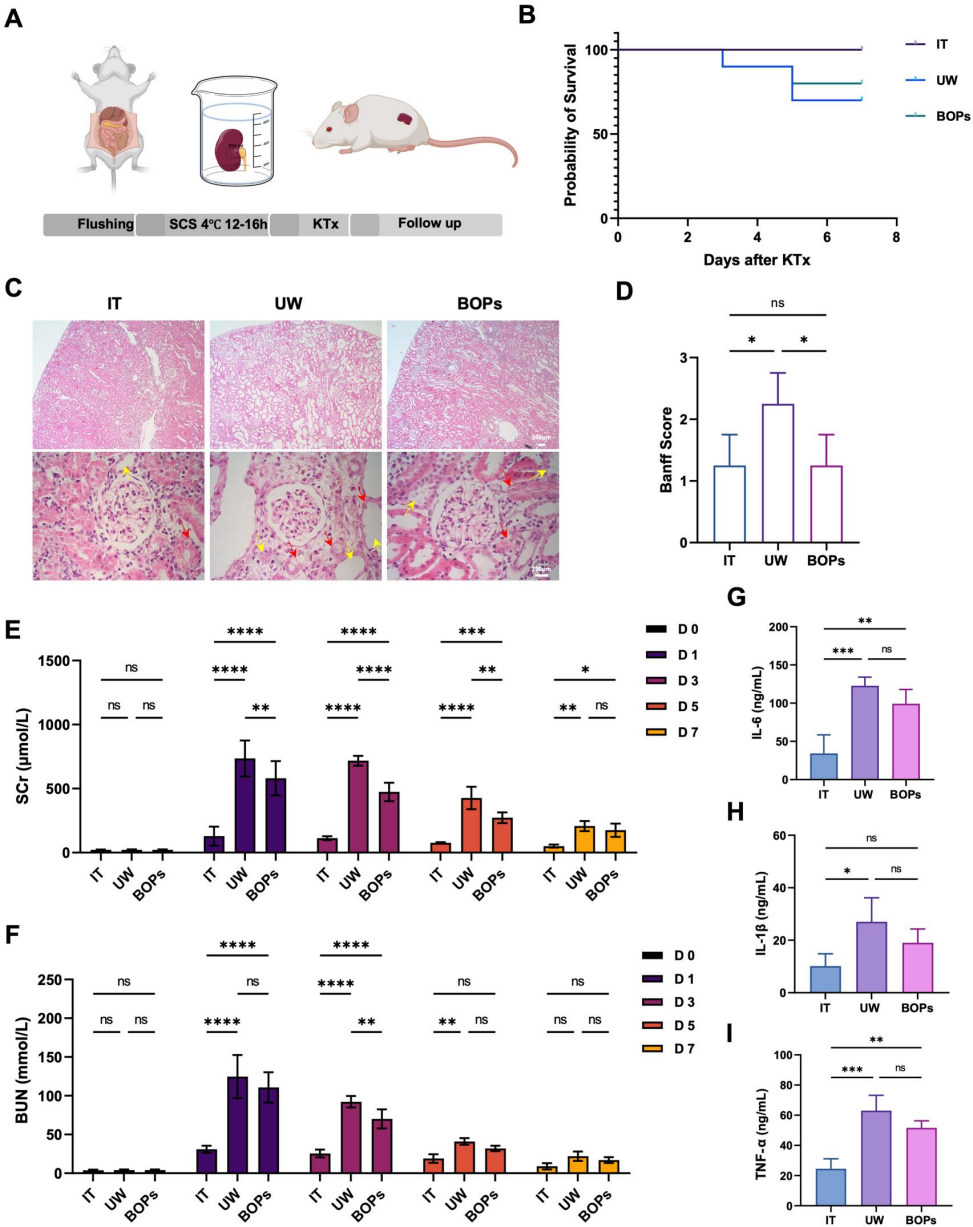
BOPs promotes rapid recovery of renal function after orthotopic kidney transplantation with static cold storage. (**A**) Schematic illustration of kidney procurement, cold preservation and transplantation procedures. (**B**) Kaplan–Meier survival curves of recipient rats after IT or cold preservation with UW solution or BOPs. (**C**) Representative H&E staining images of transplanted kidney tissues (scale bar: 200 μm). (**D**) Quantitative analysis of Banff scores. (**E** and **F**) Renal function assessment via SCr and BUN levels, measured before transplantation and on postoperative Days 1, 3, 5 and 7. (**G**–**I**) Serum levels of pro-inflammatory cytokines IL-6, IL-1β and TNF-α after transplantation. Data are presented as mean ± SD, *n* = 3. *P < 0.05, **P < 0.01, ***P < 0.001, ****P < 0.0001, ns: not significant.

Orthotopic transplantation remains the gold standard for evaluating donor kidney quality. To comprehensively assess the clinical relevance of BOPs, we performed orthotopic rat kidney transplantation with three groups: immediate transplantation (IT), cold storage in UW solution and cold storage in BOPs. Donor kidneys were preserved at 4°C for 12–16 h prior to transplantation (Figure 5A). Recipients were monitored for 7 days to assess survival and renal function. The IT group achieved 100% survival, while the UW group exhibited 30% mortality (deaths on Days 3 and 5). In the BOPs group, survival was improved relative to UW, though 20% mortality occurred (Figure 5B). Histopathological analysis revealed extensive I/R injury in the UW group, characterized by interstitial edema, tubular epithelial swelling and necrosis, brush border loss and luminal dilation. In contrast, the BOPs group displayed milder structural injury, limited interstitial expansion and relatively preserved tubular architecture, resembling kidneys in the IT group (Figure 5C and D).

Renal function recovery was further assessed by monitoring SCr and BUN. All groups exhibited postoperative SCr elevation, peaking on Day 1. The increase was most pronounced in the UW group (735.5 μmol/L), intermediate in the BOPs group (581 μmol/L) and mildest in the IT group (128.75 μmol/L). Thereafter, SCr declined progressively, with the BOPs group showing faster recovery than UW (Day 7: 175 μmol/L vs 207.5 μmol/L), though slower than IT (50.7 μmol/L) (Figure 5E). BUN showed a similar trend (Figure 5F), confirming superior functional recovery in the BOPs group. Serum cytokine levels indicated stronger systemic inflammation in cold-preserved groups compared with IT. In UW recipients, TNF-α (63.0 ng/mL), IL-6 (122.75 ng/mL) and IL-1β (22.75 ng/mL) were markedly elevated. In contrast, BOPs preserved kidneys exhibited significantly lower cytokine levels (TNF-α: 51.65 ng/mL; IL-6: 99.25 ng/mL; IL-1β: 16.75 ng/mL), suggesting reduced inflammatory activation (Figure 5G–I). Overall, BOPs effectively maintained donor kidney integrity, reduced early mortality, facilitated faster renal functional recovery and attenuated systemic inflammation after transplantation, demonstrating substantial potential as a next-generation organ preservation solution.

### The betaine component of BOPs plays a key role in reducing endothelial injury, apoptosis and improving post-transplant kidney function

**Figure 6:**
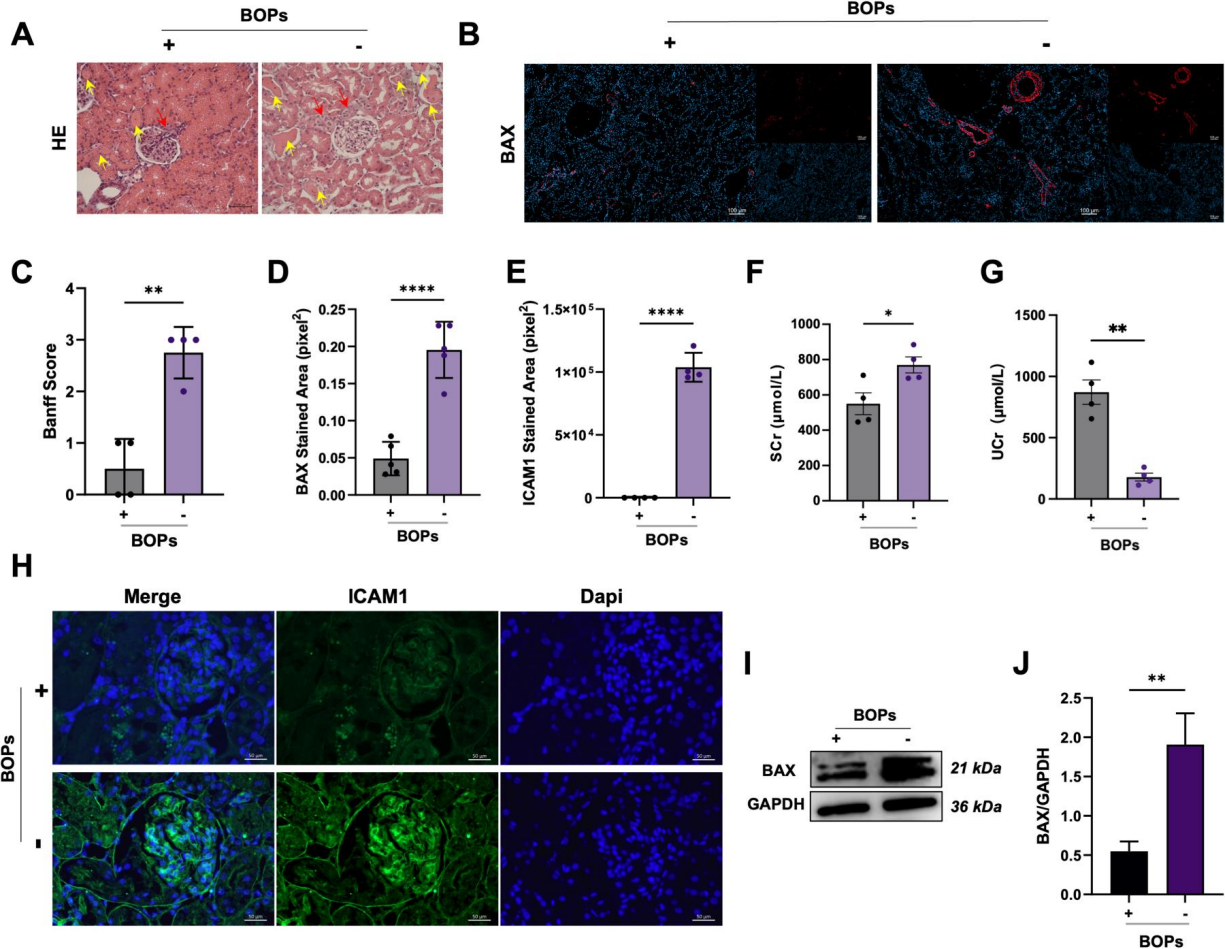
Betaine in BOPs alleviates endothelial cell injury and improves renal function after transplantation. (**A**) Representative H&E staining images of transplanted kidney tissues preserved with (+)/(−) BOPs at 2 days post-transplantation (scale bar: 100 μm). (**B**) Representative BAX immunofluorescence images of transplanted kidney tissues preserved with (+)/(−) BOPs at 2 days post-transplantation (scale bar: 100 μm). (**C**) Banff scores of transplanted kidney tissues preserved with (+)/(−) BOPs at 2 days post-transplantation. (**D**) Quantification of BAX-positive stained area (pixel^2^]. (**E**) Quantification of ICAM1-positive stained area (pixel^2^]. (**F** and **G**) Serum creatinine (SCr) and urea creatinine (UCr) levels. (**H**) Representative ICAM1 immunofluorescence images of transplanted kidney tissues preserved with (+)/(−) BOPs at 2 days post-transplantation (scale bar: 50 μm). (**I** and **J**) Immunoblot analysis of BAX protein of transplanted kidney tissues preserved with (+)/(−) BOPs at 2 days post-transplantation. Data are presented as mean ± SD, *n* = 4. *P < 0.05, **P < 0.01, ***P < 0.001, ****P < 0.0001, ns: not significant.

To further elucidate the biological role of betaine in BOPs, rat kidneys were preserved at 4°C for 12–16 h using (+) BOPs and (–) BOPs solutions. Following this, *in situ* kidney transplantation was performed, and specimens were collected 2 days post-transplant for analysis. HE staining revealed significant tubulointerstitial inflammation, tubular cast formation and mesangial cell proliferation in the (−) BOPs group (Figure 6A). The Banff scoring system for transplant pathology (Figure 6C) quantitatively confirmed higher pathological scores in the (−) BOPs group, indicating betaine’s role in reducing kidney injury. Immunofluorescence staining showed that betaine deficiency increased BAX protein expression around vascular structures (Figure 6B and D), suggesting betaine’s anti-apoptotic effects. This was supported by western blot analysis (Figure 6I and J). We also assessed ICAM1, an endothelial cell injury marker and found that (+) BOPs effectively inhibited abnormal ICAM1 protein upregulation in cold-stored transplanted kidney tissue. Renal function was evaluated by measuring serum and urinary creatinine levels (Figure 6F and G). The (+) BOPs group showed lower serum creatinine and higher urinary creatinine levels, suggesting better renal filtration. Histopathological examination confirmed that there were no pathological abnormalities in other organs such as the heart, lungs and liver after transplantation with BOPs-preserved kidneys (Figure S3), affirming the safety of BOPs application. Hematological examinations further confirmed the safety of BOPs. As shown in Figure S4, PT, APTT and TT were all within normal ranges, with most platelets maintaining a resting state (Figure S5). The elevation of FIB is recognized as a common systemic response to major surgery and trauma, aiding the body in managing I/R injury and inflammatory reactions.

## Discussion

In this study, we developed a novel cold storage solution with betaine as its key functional component and systematically evaluated its renal protective efficacy using multidimensional and multistage experimental models, including cell culture, *ex vivo* cold storage, NMP-simulated reperfusion and orthotopic kidney transplantation. Our findings demonstrated that BOPs was not only comparable to the classical UW in maintaining organ viability and function but also exhibited potential advantages in reducing apoptosis, regulating osmotic balance and promoting functional recovery. In an endothelial cell injury model, betaine effectively alleviated LPS-induced mitochondrial dysfunction, reduced intracellular ROS accumulation and suppressed the expression of pro-inflammatory cytokines (*IL-6*, *IL-1β* and *TNF-α*) as well as pro-apoptotic mediators (*BAX* and *CASP3*) [34]. The use of (+) BOPs significantly reduced pro-apoptotic protein levels in transplanted kidney tissues, suppressed ICAM1 expression in endothelial cells and effectively improved glomerular filtration function. Histological and hematological analyses verified the safety of BOPs. These results suggest that BOPs with betaine as a representative ingredient exert their protective effects primarily through preserving mitochondrial homeostasis and inhibiting oxidative stress and inflammatory cascades.

The high osmotic pressure of organ preservation solutions regulates water balance, allowing cells to resist edema, ice crystal damage and organelle dysfunction at low temperatures [35]. During cold storage, appropriate osmotic pressure maintains equilibrium between the intracellular and extracellular fluids. Excessively high or prolonged osmotic pressure, however, impairs cell membrane fluidity and damages organelles such as mitochondria and the endoplasmic reticulum. Osmotic imbalance can activate intracellular osmotic stress pathways, such as the MAPK [17] and NF-κB pathways. Notably, although BOPs and UW exhibit similar total osmolality (∼320 mOsm/kg), differences in their protective efficacy suggest that the ‘quality’ of osmolality may be more critical than its ‘quantity’. UW achieves cell dehydration via high potassium, low sodium and non-metabolic sugars (Table 1), while BOPs employs two key strategies: (i) It incorporates non-metabolic solutes such as glucuronic acid, sucrose and glycine to form a stable ‘non-metabolic osmotic pressure’, which continuously resists intracellular solute accumulation and edema caused by Na^+^ influx. These components may further reduce membrane permeability by stabilizing cell membrane structure or repairing the glycocalyx. (ii) It uses a formulation with a relatively high sodium concentration of 115 mmol/L and a moderate potassium concentration of 42 mmol/L. By reducing the intracellular-extracellular Na^+^ concentration gradient, this design aims to diminish the driving force for abnormal Na^+^ influx at low temperatures and mitigate excessive K^+^ efflux under high-K^+^ conditions—thereby avoiding osmolality reduction due to solute (K^+^) loss and more effectively controlling cell swelling. This ‘moderate dehydration’ strategy, rather than ‘forced dehydration’, may better align with the physiological needs of organs during cold storage, as evidenced by superior maintenance of cell morphology and less interstitial edema on histopathology.

In addition, the design of BOPs embodies a rethinking of the metabolic state during cold storage. Traditional preservation solutions, such as UW, aim to completely suppress metabolism, but this may lead to energy depletion, adenosine triphosphate (ATP) exhaustion, ion pump dysfunction and impaired ROS scavenging capacity—ultimately inducing apoptosis. In contrast, BOPs were supplemented with a low concentration of glucose (5 mmol/L) as a potential energy substrate. The low level of cellular metabolic activity permitted during cold preservation may induce mild cellular damage, as indicated by the elevated LDH levels observed in cells at the 0-h time point (Figure 2D). Nonetheless, the advantages of maintaining this residual metabolic activity become significantly more pronounced, especially during the critical phases of cell or tissue rewarming and reperfusion. We observed that BOPs exhibited enhanced anti-apoptotic ability after cold storage, NMP reperfusion and orthotopic transplantation. These results support our hypothesis: maintaining basal mitochondrial function and ATP levels during SCS—by moderately supplying an energy substrate to preserve mitochondrial ATP, reduce ROS generation and inhibit mitochondrial permeability transition pore opening [13, 36]—can mitigate I/R injury and apoptosis, thereby promoting graft functional recovery [37–40]. This aligns with the concept of NMP, which maintains mitochondrial activity through continuous energy supply [33, 41]. For example, in a large animal renal auto-transplantation model, Kawamura *et al.* [42] found that normothermic perfusion-preserved male pig kidneys maintained mitochondrial function and higher ATP levels; this preserved mitochondrial activity reduced post-transplant oxidative stress and inflammation, resulting in improved graft function. By adding glucose, BOPs attempts to partially mimic this ‘metabolic support’ effect under the limitations of static cold preservation, offering new potential for prolonging organ preservation time and improving graft quality.

In summary, by integrating the multiple protective functions of betaine, optimizing osmotic pressure regulation and providing moderate metabolic support, BOPs achieved effective protection of renal allografts. In small animal models, BOPs supported cold storage for up to 16 h and promoted rapid recovery of glomerular filtration function after transplantation, highlighting their promising clinical translational potential.

Despite these encouraging findings, several limitations should be acknowledged. Although we proposed potential mechanisms underlying BOPs’ superior preservation effects—including moderate cell shrinkage and optimized tissue metabolism—direct experimental evidence remains lacking. In particular, dynamic quantification of mitochondrial energy metabolism during cold storage and reperfusion injury represents a critical direction for future research. Moreover, while BOPs performed well in kidney preservation, the functional characteristics and metabolic demands of different organs vary considerably. For example, the heart, as the body’s central power organ, requires high energy turnover and is more susceptible to reperfusion injury. Whether BOPs can maintain their protective performance across organs with diverse physiological requirements warrants further investigation.

## Conclusions

In summary, this study demonstrates that BOPs significantly attenuates I/R injury and promotes graft functional recovery by inhibiting apoptosis, maintaining osmotic balance and regulating cellular metabolism during cold storage. Moreover, the betaine component provides distinct advantages by preserving mitochondrial integrity, reducing ROS production, inhibiting apoptosis and reducing endothelial inflammation. Collectively, these findings indicate that BOPs shows good organ preservation effects in cold organ preservation.

## Supplementary Material

rbag064_Supplementary_Data
