# Supplementary figures and images for: Betaine based organ preservation solution alleviates renal allograft I/R injury by protecting endothelial cells
Source: Regen Biomater. 2026 Mar 26;13:rbag064. doi: 10.1093/rb/rbag064 (PMC13091617; doi:10.1093/rb/rbag064)

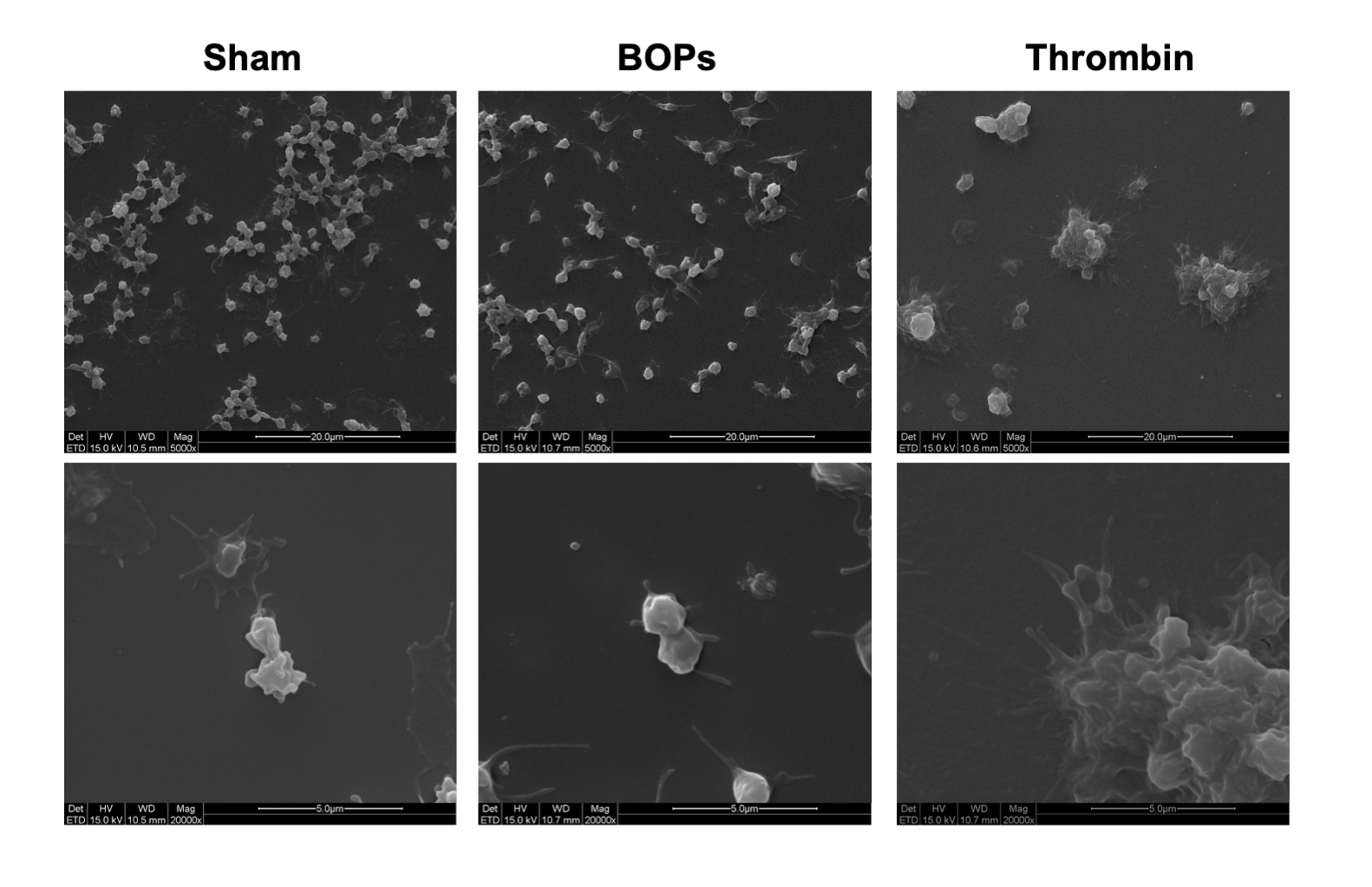

Supplement: rbag064_Supplementary_Data [file rbag064_supplementary_data.zip › fig s5.tiff]

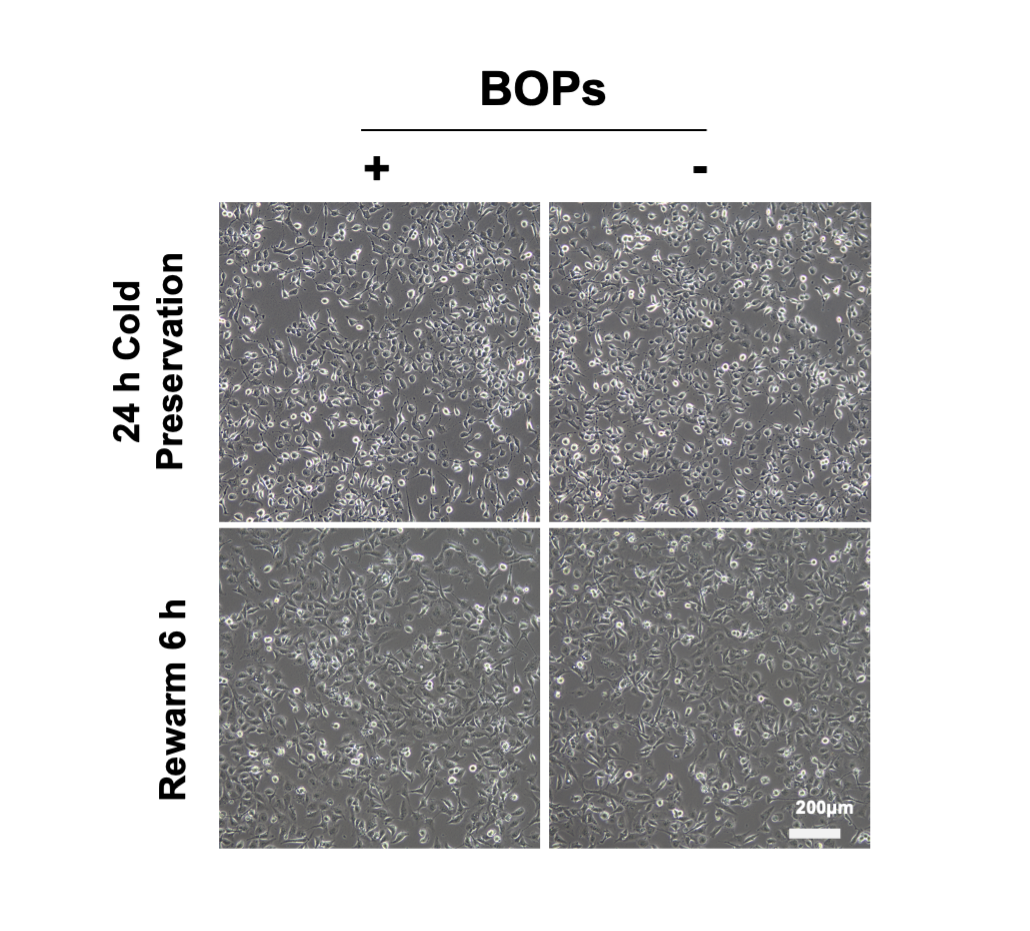

Supplement: rbag064_Supplementary_Data [file rbag064_supplementary_data.zip › fig s1.tiff]

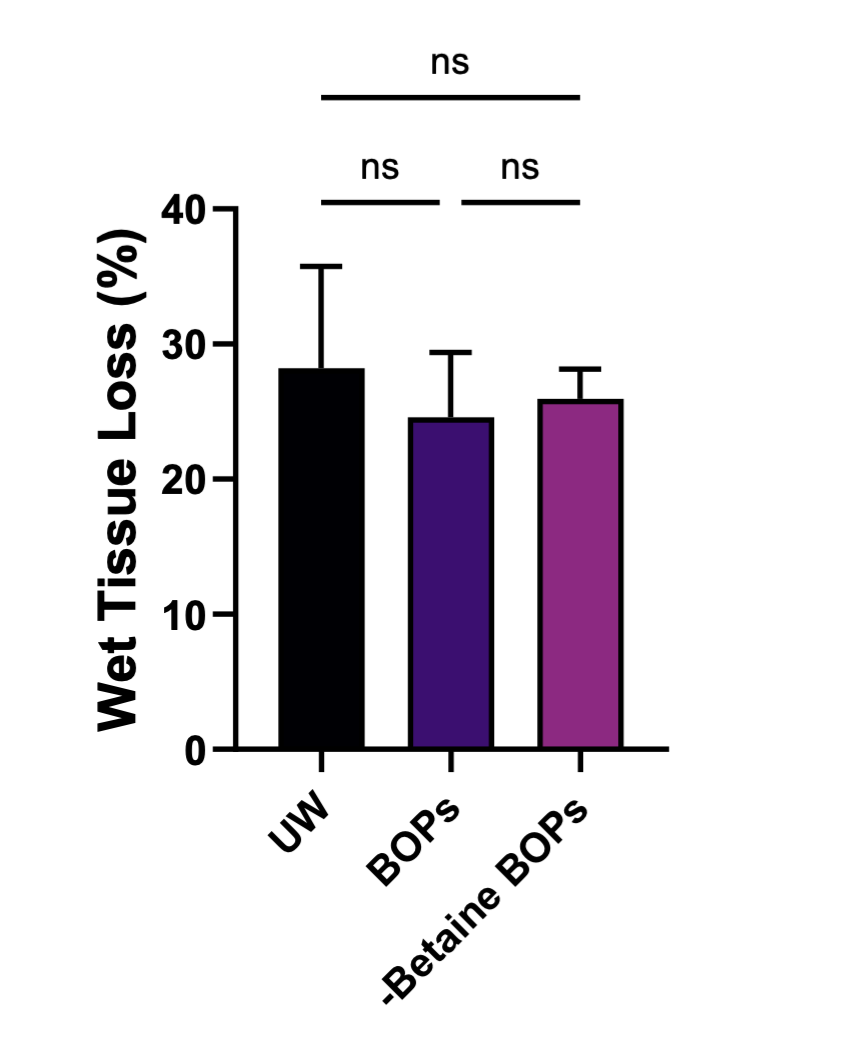

Supplement: rbag064_Supplementary_Data [file rbag064_supplementary_data.zip › fig s2.tiff]

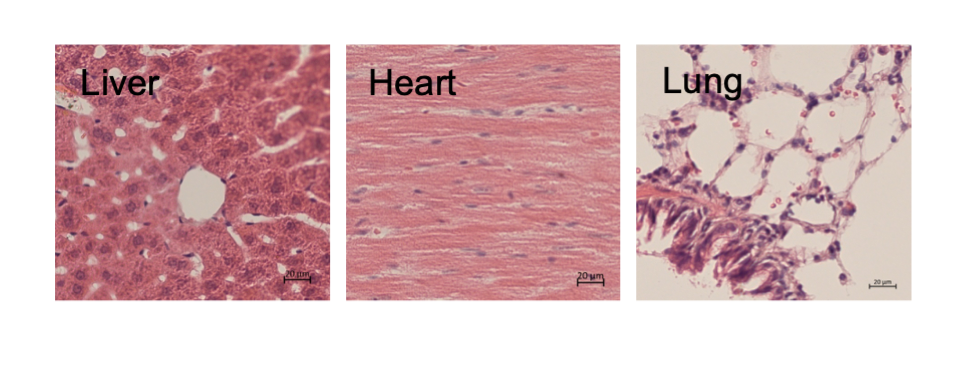

Supplement: rbag064_Supplementary_Data [file rbag064_supplementary_data.zip › fig s3.tiff]

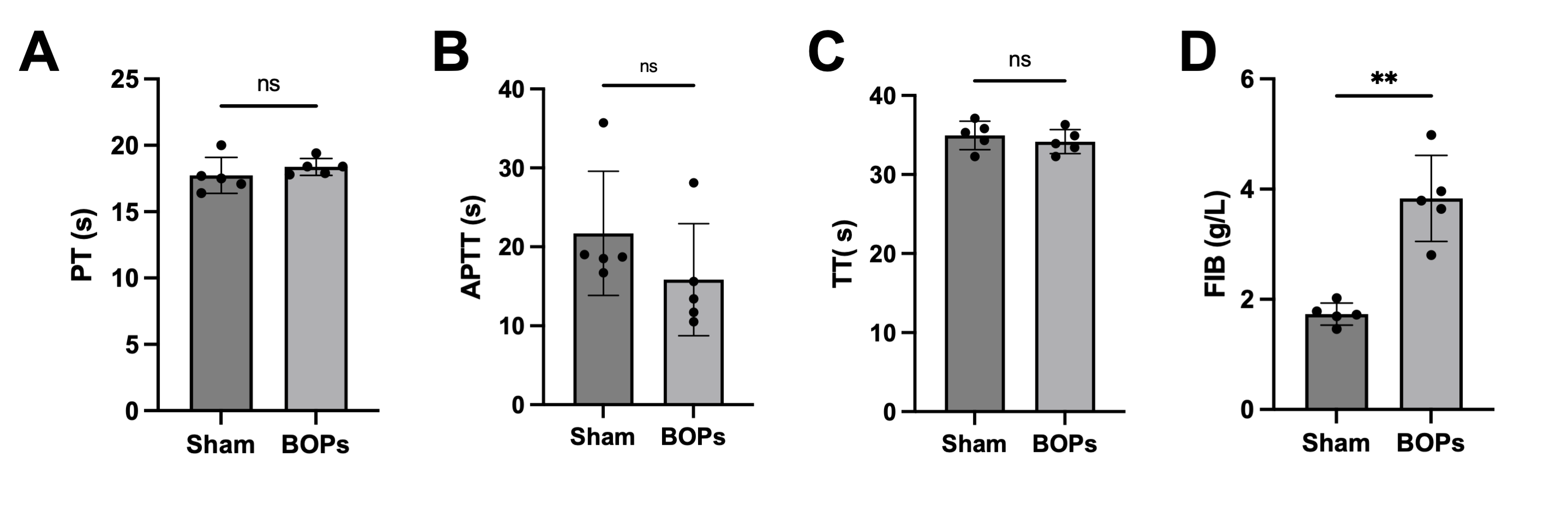

Supplement: rbag064_Supplementary_Data [file rbag064_supplementary_data.zip › fig s4.tiff]
